# Tween^®^ Preserves Enzyme Activity and Stability in PLGA Nanoparticles

**DOI:** 10.3390/nano11112946

**Published:** 2021-11-03

**Authors:** Jason Thomas Duskey, Ilaria Ottonelli, Arianna Rinaldi, Irene Parmeggiani, Barbara Zambelli, Leon Z. Wang, Robert K. Prud’homme, Maria Angela Vandelli, Giovanni Tosi, Barbara Ruozi

**Affiliations:** 1 Te.Far.T.I.-Nanotech Lab, Department of Life Sciences, University of Modena and Reggio Emilia, 41125 Modena, Italy; jasonthomas.duskey@unimore.it (J.T.D.); ilaria.ottonelli@unimore.it (I.O.); arianna.rinaldi@unimore.it (A.R.); irene.parmeggiani@unimore.it (I.P.); mariaangela.vandelli@unimore.it (M.A.V.); gtosi@unimore.it (G.T.); 2 Clinical and Experimental Medicine PhD Program, Department of Biomedical, Metabolic and Neural Sciences, University of Modena and Reggio Emilia, 41125 Modena, Italy; 3 Laboratory of Bioinorganic Chemistry, Department of Pharmacy and Biotechnology, University of Bologna, 40127 Bologna, Italy; barbara.zambelli@unibo.it; 4 Department of Chemical and Biological Engineering, Princeton University, Princeton, NJ 08544, USA; lzwang@princeton.edu (L.Z.W.); Prudhomm@princeton.edu (R.K.P.)

**Keywords:** polymeric nanoparticles, enzyme delivery, enzyme stabilization, Tween^®^ stabilization, nanomedicine

## Abstract

Enzymes, as natural and potentially long-term treatment options, have become one of the most sought-after pharmaceutical molecules to be delivered with nanoparticles (NPs); however, their instability during formulation often leads to underwhelming results. Various molecules, including the Tween^®^ polysorbate series, have demonstrated enzyme activity protection but are often used uncontrolled without optimization. Here, poly(lactic-co-glycolic) acid (PLGA) NPs loaded with β-glucosidase (β-Glu) solutions containing Tween^®^ 20, 60, or 80 were compared. Mixing the enzyme with Tween^®^ pre-formulation had no effect on particle size or physical characteristics, but increased the amount of enzyme loaded. More importantly, NPs made with Tween^®^ 20:enzyme solutions maintained significantly higher enzyme activity. Therefore, Tween^®^ 20:enzyme solutions ranging from 60:1 to 2419:1 mol:mol were further analyzed. Isothermal titration calorimetry analysis demonstrated low affinity and unquantifiable binding between Tween^®^ 20 and β-Glu. Incorporating these solutions in NPs showed no effect on size, zeta potential, or morphology. The amount of enzyme and Tween^®^ 20 in the NPs was constant for all samples, but a trend towards higher activity with higher molar rapports of Tween^®^ 20:β-Glu was observed. Finally, a burst release from NPs in the first hour with Tween^®^:β-Glu solutions was the same as free enzyme, but the enzyme remained active longer in solution. These results highlight the importance of stabilizers during NP formulation and how optimizing their use to stabilize an enzyme can help researchers design more efficient and effective enzyme loaded NPs.

## 1. Introduction

Enzymes have taken the scene as promising pharmaceutical agents to treat numerous rare and deadly diseases worldwide, including pathologies of the central nervous system (CNS) [1,2,3,4,5]. Enzyme replacement therapy (ERT), which is based on the periodic administration of specific enzymes, is currently the most suitable therapy for difficult-to-treat diseases caused by a deficit in enzymes, such as lysosomal storage disorders (LSDs) [6,7,8,9]. However, therapeutic application of enzymes is still hampered by several obstacles that limit their clinical benefits because these macromolecules frequently fail in crossing biological barriers such as the blood brain barrier (BBB), and do not reach therapeutic concentrations in the target tissues [1,4,5,10,11,12,13]. Moreover, they show immunogenicity, short half-lives in blood circulation, and a rapid loss of specific activity/therapeutic potential. This calls for extensive research in ways to protect and prolong enzyme circulation in the blood. Moreover, many of the diseases caused by a lack of a functional enzyme, such as β-glucosidase (β-Glu) in Gaucher disease, require the enzyme not only to be protected, but also to be delivered to the CNS, where the majority of the damage is done. Therefore, new therapeutic strategies are urgently required to compensate for the deficit of enzymes in order to enable their safe delivery and accumulation in diseased cells.

To overcome these problems, the idea of enzyme encapsulation into tailored nanoparticles (NPs) represents one of the most attractive strategies. Among current advances in developed NPs, polymer-based NPs have gained increasing attention as biocompatible, biodegradable, targeted, and versatile platforms [14,15,16,17,18,19,20] for the delivery of a wide array of therapeutic molecules ranging from small molecules [21,22,23,24], peptides [25,26,27,28], proteins and enzymes [2,4,29,30,31], and genetic material [32,33,34]. The encapsulation of enzymes into polymeric NPs offer several advantages compared to conventional enzyme-based therapy, including: (i) stabilizing and protecting the enzyme; (ii) improving biological activity; (iii) possibility of targeted delivery; (iv) controlling the enzyme release kinetics; (v) improving therapeutic efficacy and safety [10,35,36]. In particular, Poly(D,L-lactide-co-glycolide) (PLGA), a U.S. Food and Drug Administration (FDA)-approved polymer that can self-assemble into NPs has been of high interest [37,38,39]. This is because it is capable of encapsulating large molecules such as proteins and enzymes [40,41,42,43,44] and has also been demonstrated to have the capacity to transport large cargo across the BBB by adding targeting ligands [2,45,46,47]. This makes PLGA NPs a prime option to overcome many of these barriers for ERT treatment.

Despite these numerous advantages, the formulation process exposes the enzyme to physicochemical stresses which can alter the native conformation of the enzyme and inhibit its biological activity. For instance, the double emulsion water-oil-water technique, which is widely used for the encapsulation of enzymes into PLGA NPs, can cause protein unfolding and aggregation at the water/organic interface [48]. Various other stress conditions, including the use of organic solvents, exposure to highly energetic processes (e.g., sonication or freeze-drying), and high temperatures, frequently determine a loss of enzymatic activity. Therefore, it is necessary to develop new strategies that avoid or minimize the loss of enzyme activity during encapsulation into polymeric NPs [49,50].

One possible approach to prevent the loss of enzyme activity in solution or during formulative steps lies in the addition of stabilizers such as sugars [50,51,52,53], emulsifiers [54,55], and serum albumins [56,57,58]. Specific examples include: (1) Yun et al. used rabbit serum albumin in PLGA NPs encapsulating superoxide dismutase showing acceptable enzyme activity in vitro and in vivo [46]; (2) Osman et al. who tested PLGA NPs encapsulating DNaseI with the addition of hydroxypropyl-β-cyclodextrin (shown in other literature as an enzyme stabilizer [59]) [60]; (3) Atkins et al. that covalently linked the surfactant alkyl-glycolic acid ethoxylate to the surface of hen egg-white lysozyme to form a single enzyme NP with a surfactant shell which led to an up to 7-fold increase of enzyme activity in solution [61]; however, while offering protective features, these stabilizers can impact the physicochemical characteristics (size, morphology, and zeta potential), encapsulation efficiency of the pharmaceutic, and the pharmaceutical properties (release kinetics, biodistribution, cell uptake, etc.) of the NPs [62,63]; 4) Another work used bovine serum albumin (BSA) and its ability to stabilize β-Glu during the formulation process [56]. BSA significantly enhanced loading capacity from 6% to 30%, and was shown to quantifiably directly bind to the enzyme (studied by isothermal titration calorimetry (ITC)) forming complexes in solution. This translated to an equal stabilization of the enzyme activity due to a BSA:β-Glu enzyme complex and a higher loading content of enzyme in the NPs. Moreover, the direct complexation of the two affected release kinetics from the PLGA matrix, shifting the release from an immediate burst release over 3 h (enzyme alone) to an extended release over 6 days [56].

The polysorbates or Tween^®^ emulsifiers are another series of compounds that have been studied in the literature for their ability to influence the loading of pharmaceutics in NPs as well as for having stabilizing effects on enzymes [54,55,64]; however, there is little evidence of how the type and amount of Tween^®^ can affect enzyme stability in NPs. In this regard, understanding the effect of these variables on the NP characteristics is important to optimize the formulation strategy to increase the stability of the enzyme, and modulate its release while maintaining high activity.

This work focused on elucidating if Tween^®^ can have a stabilizing effect on a model enzyme during the formulation process into safe and biodegradable NPs. To this end, we combined β-Glu, the same model enzyme linked to the LSD Gaucher [65] used in our previous work with the stabilizer BSA [56], with Tween^®^ stabilizers and loaded them into PLGA NPs. It was determined if the type and amount of Tween^®^ (Tween^®^ 20, 60 and 80) in the enzyme solution affects the enzyme stability during NP formation.

The comparison of enzyme activity suggested that Tween^®^ 20 mixed in solution with β-Glu to form PLGA NPs led to the most promising results. Therefore, Tween^®^ 20:enzyme solutions at various molar ratios (ranging from 60:1 to 2419:1) were analyzed using ITC. After formulation of the solution mixtures into polymeric NPs, full characterization of each batch was performed to evaluate their physico-chemical characteristics, including the analysis of size, surface charge, morphology (atomic force microscopy (AFM) and scanning electron microscopy-field emission gun (SEM-FEG) analysis), loading capacity and encapsulation efficiency, and enzymatic activity. In particular, the quantity of Tween^®^ 20 and β-Glu in the PLGA NPs was measured and how it affected enzyme activity and release. Finally, the effect of combining different stabilizers was analyzed. These studies underline the importance of stabilizers to preserve high enzyme activity and possibly modulate release kinetics for improved ERT.

## 2. Materials and Methods

### 2.1. Materials

PLGA (Poly(D,L-lactide-co-glycolide), RG503H, MW ≅ 11,000) was purchased from (Evonik, Essen, Germany) and used as received. β-Glucosidase (β-Glu, MW 135 KDa), Tween^®^ 20, 60, 80 (100%, guaranteed less than 3% water), and poly(vinyl alcohol) (PVA, MW 15,000), bovine serum albumin (BSA, 66 KDa, >98% pure), dichloromethane (DCM), 4-methylumbrelliferyl-β-D-glucopyranoside, and 4-methylumbelliferone (4-MU) were purchased from Sigma-Aldrich (Milan, Italy). MilliQ water was purified by a Millipore system (Millipore, Bedford, MA, USA). Analytical grade reagents were used for all other purposes unless otherwise noted.

### 2.2. NP Formulation

Tween^®^:β-Glu mixtures were prepared by dissolving 5 mg of β-Glu with Tween^®^ and diluting to a final volume of 500 µL with MilliQ. To analyze the various Tween^®^ s, the molar ratio of Tween^®^:β-Glu was held constant at 2248:1 (corresponding to 20% *v/v* in water). Tween^®^ 20:β-Glu solutions were formed similarly using different molar rapports of Tween^®^ 20 to β-Glu to create solution 1–4, with solution 0 being a control of β-Glu without Tween^®^ 20 (Table 1).

In the case of PLGA encapsulating BSA:β-Glu or Tween^®^ 20:BSA:β-Glu solutions, the same procedure was followed but with the addition of 50 mg of BSA (20:1 BSA:β-Glu molar ratio) in the first aqueous phase before the double emulsion formulation.

PLGA NPs were produced utilizing the double emulsion method (w1/o/w2) [56,66]. The preformed Tween^®^:β-Glu solutions (Table 1) were added to the organic polymer solution (50 mg of PLGA in 2.5 mL of DCM) and the first emulsion (w/o) was obtained by sonicating on ice (amplitude 54%, 80 W) for 45 s using a probe sonicator (SLPe, Branson, Milan, Italy). The first emulsion was added to 8 mL of a 1% (*w/v*) PVA and sonicated on ice (80 W for 45 s) to obtain the final w1/o/w2 emulsion.

All formulations were mechanically stirred (RW 20 DZM, Janke & Kunkel, IKA Lab, Sigma Aldrich, Milan, Italy), at 1400 rpm, for 2 h at room temperature (RT) to evaporate the organic solvent. Finally, the NPs were purified by centrifugation (Multispeed Centrifuge PK 121, ALC, Bodanchimica, Cagliari, Italy) at 9,700 rpm for 10 min to remove the residual PVA and enzyme from the solution. The supernatant was discarded and the NPs were resuspended in 4 mL MilliQ water with the aid of a Vortex mixer (Velp Scientifica, Monza Brianza, Italy) and sonication bath (30 s cycles until complete resuspension). The percent yield was calculated by lyophilizing 0.5 mL of the resuspended NPs (LyoLab 3000, Heto, Milan, Italy) and weighing the amount of NPs recovered. The lyophilized NPs were also used to extract and quantify the amount of Tween^®^ and enzyme loaded into the NPs (See Methods 2.6–2.8). The remaining NP suspension was aliquoted in 1 mL samples and stored at 4 °C.

### 2.3. ITC Analysis

As previously published [56], the possible interaction between Tween^®^ 20 and β-Glu was performed by ITC (25 °C), using a high-sensitivity VP-isothermal titration calorimetry microcalorimeter (Malvern Panalytical, Malvern, UK). 50 mg of β-Glu were solubilized in phosphate buffer saline (PBS, pH 6, 37 µM). 0.5 mM Tween^®^ was passed on a pre-equilibrated PD SpinTrap G-25 (GE Healthcare, Chicago, IL, USA) and the reference cell was filled with MilliQ water. The Tween^®^ (13% *w/v*) was eluted and diluted to 1 mM with PBS and loaded into the ITC syringe. 1–2 μL of the Tween^®^ water solution were injected and discarded from the data at the beginning of each experiment to avoid artifacts in the injection port during equilibration. Once stabilized, the first addition was added. Each titration consisted of an injection of 10 μL Tween^®^ (0.5 mM) into a 37 μM β-Glu solution via a computer-controlled 310 μL microsyringe. A 300 s delay was incorporated to allow for equilibration between titration injections. Titrations of Tween^®^ 20 into PBS or PBS into β-Glu solutions were used as controls. Integrated heat data were fitted using a nonlinear least-squares minimization algorithm to a theoretical titration curve, using AFFINImeter (V2.1802.5, Edificio Emprendia, Campus Vida, Campostela, Spain) through the independent sites approach for each titration. The thermodynamic fitting parameters consisted of Δ*H* (cal mol^−1^ change in reaction enthalpy), *N* (stoichiometry), and *KA* (M^−1^, binding constant). The parameter *Qdil* (heat of dilution, J mol^−1^) was also adjusted as a fitting parameter. The relationships Δ*G* = −*RT*ln*KA* (*R* = 8.314 J^−1^ mol^−1^ K^−1^, *T* = 298 K) and Δ*G* = Δ*H* − TΔ*S* were used to calculate the reaction entropy. Goodness of fit (GoF) was used to evaluate the reliability of the obtained fits based on software provided parameters.

### 2.4. Size and Zeta Potential Analysis

The mean particle size (Z-Average) and polydispersity index (PDI) of all samples were determined by photon correlation spectroscopy (PCS) analysis, using a Zetasizer Nano ZS (Malvern, Milan, Italy; laser 4 mW He–Ne, 633 nm, laser attenuator automatic, transmission 100–0.0003%, detector avalanche photodiode, Q.E. > 50% at 633 nm, T = 25 °C).

All samples were diluted before being analyzed: 10 µL of purified NPs suspension were diluted with MilliQ water to 1 mL to arrive at a final concentration of ~0.1 mg/mL. At least three individual NP formulations were prepared and analyzed to yield a mean for each data set.

The zeta potential (ζ-pot) was measured using a Zetasizer Nano ZS (Malvern, Milan, Italy) with a combination of laser doppler velocimetry and a patented phase analysis light scattering method (M3-PALS). The same samples subjected to PCS (0.1 mg/mL) were analyzed using DTS1070 ζ-pot cuvettes and expressed as the mean of at least three individual NP preparations.

### 2.5. Microscopy Analysis

Atomic force microscopy (AFM, Park Instruments, Sunnyvale, CA, USA) was used to evaluate the morphology of NPs formulated with Tween^®^:enzyme solutions. Analyses were performed using triangular silicon tips at 25 °C in air and in non-contact mode. The cantilever resonant frequencies were set at ~160 kHz. Water-diluted NPs (20 μL, ~0.01 mg/mL) were added to a small mica disk (1 cm × 1 cm), let dry for 2 min and the excess solution was removed. The topographical images were flattened using second-order fitting to remove sample tilt.

The same samples were also imaged by scanning electron microscopy field emission gun (SEM-FEG). Briefly, a drop of a water-diluted suspension of the samples (about 0.01 mg/mL) was placed on a 200-mesh copper grid (TABB Laboratories Equipment, Berks, UK), allowed to adsorb, and the suspension surplus was removed by filter paper. All grids were analyzed using a Nova NanoSEM 450 (FEI, Hillsboro, OR, USA) electron microscope operating at 30 kV using a SEM II detector in field-free mode.

### 2.6. Weight Yield

Purified NPs (0.5 mL) were freeze-dried and weighed (−60 °C, 1 × 10^−3^ mm/Hg for 8 h; LyoLab 3000, Heto-Holten, Allerod, Denmark), and the yield% was calculated as follows:

(1) $$\mathrm{Yield}\left(\%\right)=\frac{\mathrm{freeze}-\mathrm{dried}\;\mathrm{sample}\;\left(\mathrm{mg}\right)}{\mathrm{PLGA}\;\left(\mathrm{mg}\right)+\mathrm{enzyme}\;\mathrm{used}\;\mathrm{for}\;\mathrm{preparation}\left(\mathrm{mg}\right)}\times 100$$

### 2.7. Quantification of β-Glu in PLGA NPs

To quantify the β-Glu encapsulated in the NPs, a volume of 0.5 mL of purified NP suspension was freeze-dried, weighed, added with DCM (0.25 mL) and subjected to agitation with Vortex mixing (ZX4 Advanced IR Vortex Mixer, Velp, Usmate, Italy) for 30 s to dissolve the PLGA. Then, the enzyme was extracted using 250 μL MilliQ in which the PLGA is insoluble. These samples were centrifuged (Spectrafuge 24D Labnet International Inc., Edison, NJ, USA) at 10,000 rpm for 2 min, to properly separate the aqueous from organic phase. 50 µL of the aqueous phase containing the enzyme were injected and quantified by reverse-phase high performance liquid chromatography (RP-HPLC) (Figure S1A).

As previously described [56] the HPLC (JASCO Europe, Cremella, Italy) was comprised of a Model PU-2089 Plus pump and a 50 μL sample loop (Jasco, Model 7725i) and fitted with an AerisTM C8 analytical column (3.6 μm WIDEPORE XB-C8 200 Å, Phenomenex, Bologna, Italy). The gradient consisted of a biphasic system using A [0.1% *v/v* trifluoroacetic acid in MilliQ water (pH~2)] and B [0.1% *v/v* trifluoroacetic acid in acetonitrile] where B was increased from 20 to 80% over 6 min (1.2 mL/min) under isothermal conditions at 70 °C (model 7971 Column Heater, Jones Chromatography, Rheinfelden, Germany). The absorbance was monitored at 210 nm using a UV detector (Jasco UV-1575, Carpi, Italy). The peak area was integrated, and a standard curve ranging from 20 to 1600 μg/mL (*y* = 40538*x* + 4330.9, *R*^2^ = 0.99) was used to calculate the β-Glu concentration.

The entrapment efficiency (EE%) and the loading capacity (LC%) were calculated as follows:

(2) $$\mathrm{EE}\%=\frac{{\beta \mathrm{Glu}}_{\left(\mathrm{NPs}\right)}}{{\beta \mathrm{Glu}}_{\left(\mathrm{total}\right)}}\times 100$$

(3) $$\mathrm{LC}\%=\frac{{\beta \mathrm{Glu}}_{\left(\mathrm{NPs}\right)}}{{\mathrm{NP}}_{\left(\mathrm{total}\right)}}\times 100$$

where βGlu_(NPs)_ indicates the amount (mg) of βGlu loaded in the NPs, βGlu_(total)_ refers to the amount (mg) of β-Glu originally used in the formulation, and NP_(total)_ the amount (mg) of NPs recovered. All data were calculated using three individual formulations for each sample type.

### 2.8. Quantification of Tween^®^ in PLGA NPs

To determine the amount of Tween^®^ 20 incorporated into or absorbed onto the NPs, the Tween^®^ 20 was extracted from the NPs and analyzed by HPLC-evaporative light scattering detector (ELSD) analysis by modifying a previously published protocol [67].

A weighed amount of NPs (about 10 mg) was dissolved in 1 mL of DCM and vortexed for 20 s to solubilize the PLGA. To extract Tween^®^ 20, 1 mL of MilliQ water was added to the DCM suspension and vortexed again. Samples were stirred (Multistirrer, Magnetic Stirrer Velp Scientifica, Monza Brianza, Italy) to evaporate the DCM, and precipitate the PLGA. The volume of the resulting suspension was adjusted to 1.5 mL with MilliQ water and centrifuged (Spectrafuge 24D, Edison, NJ, USA) at 13,000 rpm for 3 min to pellet the PLGA. The supernatant was then analyzed by ELSD-HPLC (20 µL injection volume) (Figure S1B). Tween^®^ 20 quantification was carried out on a 1260 Infinity II HPLC system (Agilent, Milano, Italy) with a fixed flow rate of 1.2 mL/min at RT, coupled with two detectors: (1) a UV detector (1260 variable wavelength detector (VWD)) set at λ = 210 nm; (2) an ELSD system 1260 Infinity II (settings: evap. = 45 °C; neb. = 45 °C; gas = 1.60 SLM). A C8 (Phenomenex^®^) analytical column was used, and elution was obtained using a gradient consisting of A [MilliQ water] and B [acetonitrile]. The mobile phase gradient was optimized as follows:

- 0–5 min: 0–0% B
- 5–10 min: 0–60% B
- 10–15 min: 60–80% B
- 15–17 min: 80–0% B

A calibration curve was obtained using the same methodology (*y* = 0.101*x* − 1.1858, *R*^2^ = 0.9932), and linearity was achieved from 2.5 to 15 µg injected in 20 µL.

Tween^®^ 20 content and the mol:mol ratio Tween^®^ 20:β-Glu were calculated as follows:

(4) $${\mathrm{Tween}}^{®}20\;\mathrm{Content}=\frac{\mathrm{mg}\;{\mathrm{Tween}}^{®}20}{\mathrm{mg}\;\mathrm{NPs}}\times 100$$

(5) $$\mathrm{molar}\;\mathrm{ratio}\;{\mathrm{Tween}}^{®}20:\beta -\mathrm{Glu}=\frac{\mathrm{mol}\;{\mathrm{Tween}}^{®}20\;\mathrm{in}\;\mathrm{the}\;\mathrm{NPs}}{\mathrm{mol}\;\mathrm{of}\;\mathrm{enzyme}\;\mathrm{in}\;\mathrm{NPs}}$$

### 2.9. β-Glu Activity Assay

The activity of β-Glu was assessed using the substrate 4-methylumbrelliferyl-β-D-glucopyranoside. One hundred µL containing 45 µL of NPs or supernatant, 25 µL 4× McIlvain buffer pH 6 (0.4 M citric acid, 0.8 M monobasic phosphate), 5 µL water MilliQ, and 25 µL 4-methylumbrelliferyl-β-D-glucopyranoside (2 mg/mL) substrate were incubated for 1 h in a 96-well plate under agitation at 170 rpm and heated to 37 °C. Each sample underwent a 1:50 dilution with MilliQ water and the reaction was stopped by adding 10 µL to 190 µL of glycine “stop buffer” solution (250 mM, pH 10.7) in a separate 96-well plate; the enzymatic product (4-MU) was measured by a fluorimeter (Synergy HTX Multi-Mode Reader, BioTek, Winooski, VT, USA) ex:em 365:488 nm. β-Glu activity was expressed as pmol of the product, 4 MU, per hour per µg of enzyme. Quantification was calculated using an internal standard curve produced on the same 96-well plate using 0, 2, 5, 10, 20, 50, 100 μL of a known 4MU solution (5 µM) diluted in “stop buffer” (250 mM glycine, pH 10.7) to a final volume of 200 µL. Control samples included NP resuspension buffer (MilliQ water), as well as empty NPs (NPs formulated without enzyme), Tween^®^ solutions at the concentrations found in the NPs, and BSA solutions equal to those present in the NPs. These controls did not show an increased background compared to the water sample alone and were not considered.

### 2.10. Enzyme Release Assay

The effect of Tween^®^ 20 on the release profile and activity of β-Glu from the PLGA NPs was analyzed in two different biologically relevant conditions: pH 7.4 (PBS) to simulate systemic release and pH 4.5 (acetate buffer) to simulate the lysosomal environment. Individual samples for each time point of PLGA NPs encapsulating β-Glu or Tween^®^ 20:β-Glu solutions were analyzed from 1 h to 14 days at each pH. NP suspensions (0.9 mL) were buffered with 0.1 mL of release buffer (PBS 10× or acetate buffer 10x) in Eppendorf tubes (final volume 1 mL in 1× concentrated buffer). Samples were incubated in a heated bath (ISCO GTR 2000, Optolab, Concordia Sulla Secchia, Italy) at 37 °C under electromagnetic stirring. At each time point (1 h, 3 h, 6 h, 24 h, 72 h, 7 days, and 14 days) corresponding samples were centrifuged (Spectrafuge 24D centrifuge, Edison, NJ, USA) at 13,300 rpm for 7 min in order to separate NPs as a pellet. An aliquot of the supernatant (45 µL) containing the released enzyme was tested for enzyme activity. The remainder (960 µL) was freeze-dried, re-suspended with 0.1 mL MilliQ water (with the aid of a vortex mixer) and quantified by RP-HPLC analysis as previously described.

### 2.11. Statistical Analysis

Statistical analysis in all assays was performed using the Student *t* Test where * *p* < 0.05 and ** *p* < 0.01 as indicated in each figure. All statistical analyses (Z-Average, ζ-pot, PDI, %EE, enzyme activity and release) were performed in triplicate on three independent samples or NP formulation (N = 3) and the standard deviation (S.D.) from the mean of the three separate samples is represented by a “±” or error bars.

## 3. Results

Tween^®^ is a trademarked line of surfactants which have been shown in the literature to help NP formation and stabilize enzymes [54,55,64,68,69,70]. Therefore, an initial study was performed to compare the formulation of the model enzyme β-Glu mixed in solution with the Tween^®^ series of surfactants, which differ in their chemical structure, as their sorbitan-derived core is esterified respectively with lauric (Tween^®^ 20), stearic (Tween^®^ 60), or monooleic acid (Tween^®^ 80). The aim was to determine which variant poses the highest potential for formulating and preserving enzyme activity in PLGA NPs. β-Glu mixed in solution with each different Tween^®^ in the aqueous phase before formulation with the double emulsion method did not produce any difference in size or ζ-pot of the NPs compared to empty PLGA NPs or those containing free β-Glu, resulting unanimously with monodisperse particles ranging from 177 to 208 nm with low PDI < 0.2 and a negative surface charge of the NPs ranging from −16 to −23 mV (Table 2).

**Figure 1:**
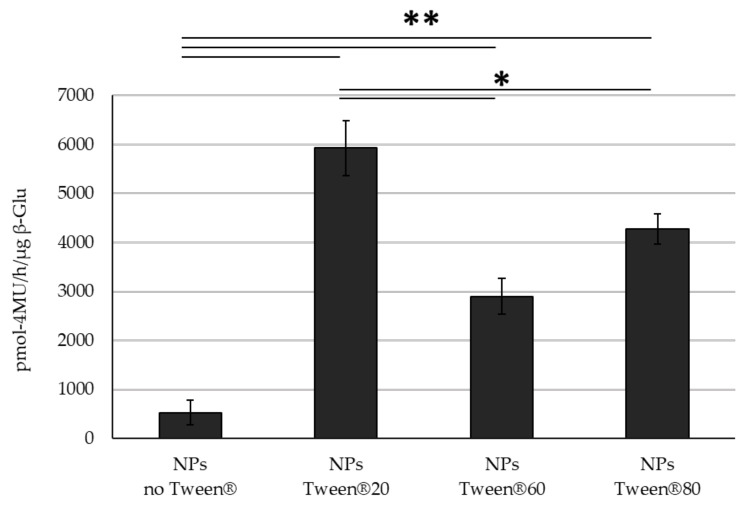
Activity Comparison. Activity of β-Glu mixed with Tween^®^ 20, 60, 80 and encapsulated into PLGA NPs. Statistical analysis was performed using the Student *t* test where * *p* < 0.05, ** *p* < 0.01 and N = 3 individual NP formulations for each type.

The fact that the different Tween^®^s had little effect on the NP self-assembly process is not surprising, given that they have previously been used in the literature for this purpose. Further analyzing the NP characteristics, varying the type of Tween^®^ did not affect the %yield of the NPs; however, statistical analysis showed that while each Tween^®^ was not significantly different from each other (*p* = 0.23), even though all three showed slightly improved %EE compared to control NPs with only β-Glu, only NPs containing Tween^®^ 20 were significantly higher than the ones without Tween^®^ (3.9 and 7.8 respectively, *p* = 0.049) (Table 2). Similarly to the increase in %EE a higher enzyme activity was also observed. All NPs with Tween^®^:β-Glu significantly outperformed the control NPs which contained β-Glu without Tween^®^. Interestingly, while the %EE was not significantly different between NPs with the different Tweens^®^, the enzyme activity did show variation. Here it is important to note that activity of β-Glu within the intact NPs was quantified to avoid using organic solvents that could potentially damage the enzyme during extraction; however, control samples showed that PLGA did not interfere with a direct comparison of NPs containing enzyme with or without Tween^®^. In this case, the presence of Tween^®^ 20 in the enzyme solution led to NPs that statistically outperformed its other Tween^®^ counterparts in terms of preserved enzyme activity (Figure 1). These results closely resemble the protective effect of Tween^®^ 20 on β-Glu when disposed to the same stresses (organic solvent, sonication, and centrifugation) without the presence of PLGA. While Tween^®^ 20 has no effect on the free enzyme, it stabilizes the enzyme when stressed leading to retention of ~40% of its activity compared to only 10% for the un-stabilized enzyme under stress (Figure S2). Because the major barrier to effective ERT is delivering a therapeutically relevant amount of enzyme to the diseased site, the improved %EE and activity of Tween^®^ 20 merit further studies to better understand the correlation between Tween^®^ 20 and enzyme stabilization and therefore only Tween^®^ 20 was chosen for more in depth studies.

Due to the improved features of Tween^®^ 20:β-Glu solution mixtures in the formulation of PLGA NPs, a more detailed study of the solution and its incorporation into the NPs during formulation was performed. To this end, 5 different Tween^®^:β-Glu solutions (Solutions 0–5) were analyzed ranging in a mol:mol ratio of 60–2419:1, where solution 0 was designated as a control with no Tween^®^ but only β-Glu.

The total volume of all mixtures and the amount of enzyme were held constant at 500 µL and 5 mg respectively (solution compositions are described more in detail in Methods Section 2.2 (Table 1)). Contrary to the previously published results using BSA:β-Glu complexes [56] that showed small nanosized structures upon complexation with a range from ~9–60 nm, Tween^®^:β-Glu mixtures analyzed by PCS showed erratic results due to the non-homogeneity of the sample, as highlighted by PDI values ranging from 0.35 to 0.80, suggesting poor or uncontrolled complexation. Here the ζ-pot reached neutral values around 2 ± 2 mV. (Table 3).

**Figure 2:**
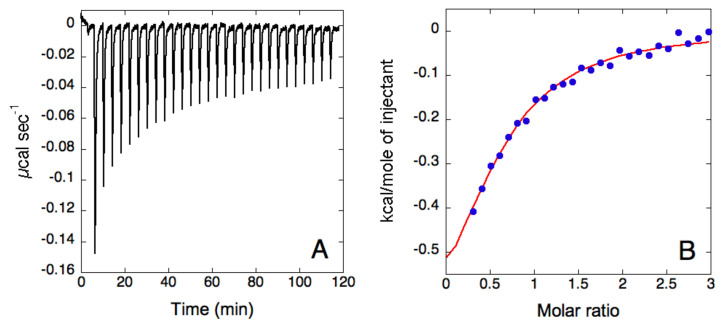
Isothermal titration calorimetry analysis of Tween^®^ 20 and β-glucosidase. (**A**) Raw titration data of Tween^®^ 20 (0.5 mM) titrated over β-Glu (37 µM) in PBS buffer, pH 6. (**B**) Binding isotherm of Tween^®^ 20 titration over β-Glu, obtained by integrating raw data for the protein titration. The blue dots represent the experimental data and the red curve represents the fit of the data using a single set of sites model.

This unquantifiable binding between Tween^®^ 20 and β-Glu was also supported by ITC. In this study, Tween^®^ 20 (0.5 mM) was titrated over β-Glu (37 μM) (Figure 2). Similar to the PCS analyses, ITC suggested a weak binding interaction with the fit of the data indicating the presence of a single binding event with N = 0.62 ± 0.08 Ka = 6 ± 1 × 104, ΔH = 3.8 ± 0.4 kJ mol^−1^, ΔS = 79.9 J mol^−1^ K^−1^. The relatively low binding constant provided a c value of 1, lower than the optimal one (c = M × KA × N), which indicated that the conditions used could provide only a suboptimal sigmoidicity of the binding isotherm, without a clear inflection point. In theory, the value of “c” could be improved by increasing the enzyme and surfactant concentrations. However, this was not a possibility in this case as doubling the Tween^®^ 20 concentration in the titration syringe led to a high heat of dilution for the detergent, which increased the signal-to-noise-ratio and masked the heat of binding. For this reason, the stoichiometry of the reaction could not be reliably derived from the fit under the conditions used. In the hypothesis of a 1:1 binding event, the dissociation constant can be calculated as 17 ± 3 µM. The derived thermodynamic signature shows both favorable enthalpic and entropic values, with a prevalence of the entropic term. This is likely due to the hydrophobic nature of the interaction between the detergent and the enzyme. Hydrophobic interactions are indeed usually entropy-driven, as water molecules, previously organized around the hydrophobic surfaces, are released into the bulk of the solution. It is important to note that during titration, the Tween^®^ 20 concentration in the sample cell is lower than the critical micellar concentration (cmc), indicating that it represents the enzyme interaction with single molecules of the detergent.

The ITC data of Tween^®^ 20:β-Glu solution mixtures suggested that the stabilizing effect did not arrive from a strong quantifiable complexation with the enzyme β-Glu, but from an effect during the formulation of the NPs. This also supports the almost neutral zeta potential with large errors along with the lack of distinct nano sized self-assemblies in solution. To better evaluate the stabilizing effect of Tween^®^ 20 on the enzyme each solution mixture was used to formulate β-Glu-PLGA NPs and were characterized for Z-Average, ζ-pot, %yield, %LC and %EE (Table 4).

As expected based on the initial study, varying the molar rapport of Tween^®^ 20: β-Glu had no significant effect on the physico-chemical characteristics of the NPs. Each NP formulation had a comparable Z-Average, PDI, and ζ-pot (~190 nm, PDI < 0.1, and −18 mV) as that without stabilizer. There was a slight difference in %EE among the samples, with Solution 2 showing the highest encapsulation at 11.1%; however, the %EE was statistically higher for NPs formulated with all of solution mixtures when compared to the control sample without Tween^®^ even though the differences between each mixture were not statistically significant.

**Figure 3:**
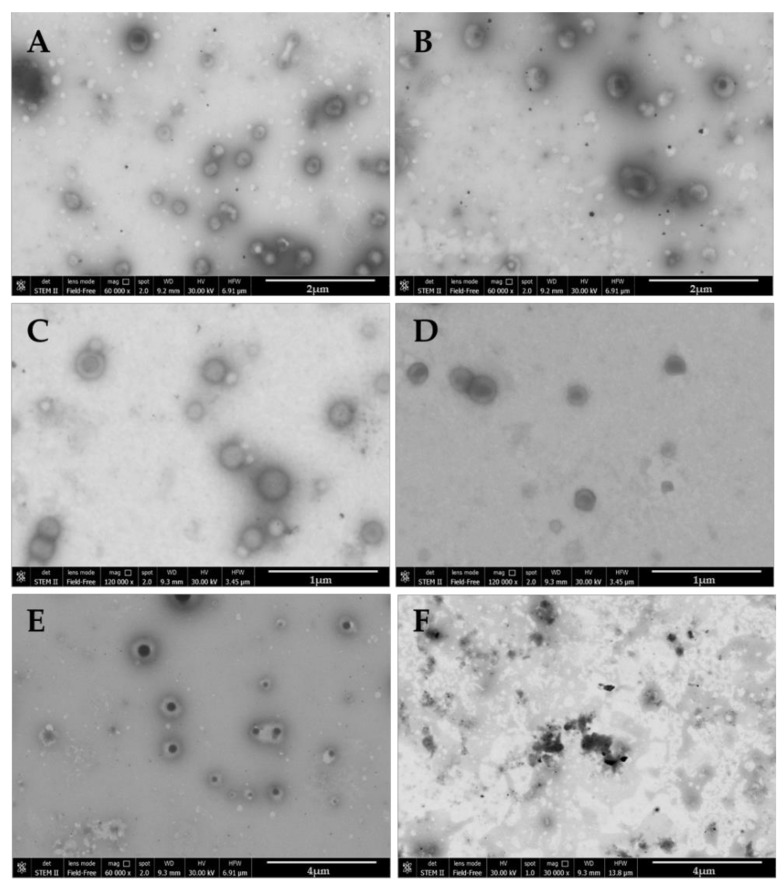
SEM-FEG Nanoparticle images; (**A**) NPs PLGA:Solution0; (**B**) NPs PLGA:Solution1, (**C**) NPs PLGA:Solution2; (**D**) NPs PLGA:Solution3; (**E**) NPs PLGA:Solution4; (**F**) Solution Tween^®^ 20: β-Glu non-formulated. Note: all formulations contain 5 mg β-Glu.

To further analyze the morphology and self-assembly of each NP, AFM and SEM-FEG analysis were performed (Figure 3 and Figure S3). AFM analysis demonstrated that all Tween^®^ 20:enzyme solutions were incorporated into NPs ranging from 150 to 300 nm with some aggregates reaching 500 nm, as seen with NPs encapsulating Solution 3. Analyzing the images, it was seen that with the higher amount of Tween^®^ (Solution 4) more uniform spherical NPs were formed (Figure S1).

SEM-FEG analysis supported these findings where all images showed spherical NPs ranging from 150 to 300 nm. With this microscopic method, however, the double emulsion containing Tween^®^ is more apparent. Increasing the amount of Tween^®^ led to the NPs becoming more uniform and presenting a halo around the denser core (Figure 3E). Further supporting the ITC data, Solution 4 was analyzed in solution pre-formulation and no self-assemblies were identifiable (Figure 3F).

To determine if these structural differences were caused directly by a variation in the amount of Tween^®^ 20 incorporated into the NPs, a quantification of Tween^®^ 20 content was performed by ELSD-HPLC, following a protocol modified from a previously published article (Table 5) [67]. While the HPLC-ELSD technique allowed for the quantification of the Tween^®^ in the NPs, reverse phase columns are incompatible with the polymer PLGA, meaning no structural information could be provided of intact NPs. Therefore, Tween^®^ 20 was extracted from the NPs using an organic solvent, to destroy the 3D-assembly, and an aqueous solvent was used to solubilize and quantify the Tween^®^ to determine if there is a correlation between the physical characteristics and the presence of Tween^®^ 20. Interestingly, the amount of total Tween^®^ 20 incorporated into the NPs remained constant independently from the Tweeen^®^ 20:enzyme solution mixture encapsulated, despite the increased amount of surfactant in the initial aqueous solution (~9 mg/100 mg NPs). This is an interesting result because even over such large differences in the amount of Tween^®^ 20 in the aqueous phase (0.5–20% *v/v*), there appeared to be a saturation effect where the PLGA NPs have an incorporation limit of ~ 10% of the total mass of the NPs. This means, the only difference in final Tween^®^ 20 amount can be observed in the ratio of Tween^®^:β-Glu (increasing from 841:1 to 1560:1) due to the difference in %EE and final %yield.

**Figure 4:**
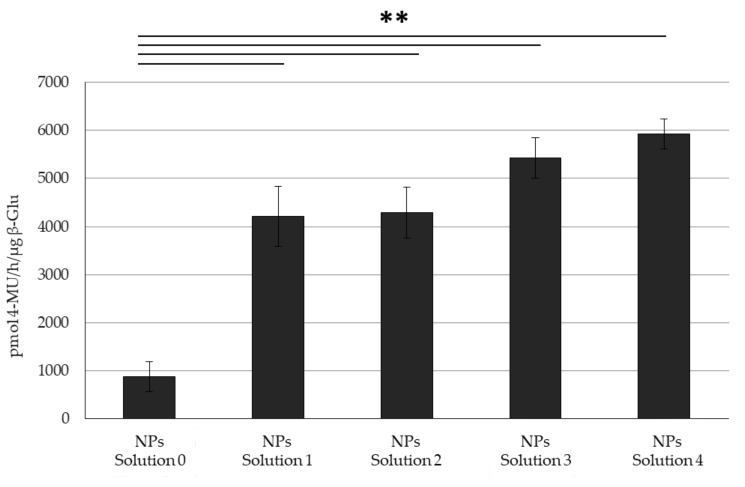
Activity Comparison. Activity of Tween^®^ 20:β-Glu solutions at different molar ratios (Solutions 1-4) and encapsulated into PLGA NPs. Statistical analysis was performed using the Student *t* test where ** *p* < 0.01 and N = 3 individual NP formulations of each type.

While the increased amount of Tween^®^ 20 appeared to form more homogenous NPs according to the microscopic analysis, the constant PCS results, and amount of Tween^®^ 20 in the NPs was directly translated to the activity in the NPs. While all Tween^®^ 20:enzyme solution mixtures yielded NPs with higher activity respective to those without Tween^®^ 20, the difference between the various mixtures was insignificant, showing only a slight trend towards higher activity correlated to a higher amount of Tween^®^ 20 in the initial solution, as well as the trend towards higher Tween^®^ 20:β-Glu molar rapports (Figure 4, Table 5).

While interesting, this brings up another question as to why, even when the amount of residual Tween^®^ 20 remains constant (~9.5%) in the final composition of NPs, the trend towards a higher molar rapport of Tween^®^ 20:β-Glu also led to a trend towards higher activity. One possible conclusion would be that the protective effect is completely ascribed to stabilization from the stresses in solution. To study if the presence of Tween^®^ 20 affects the release and activity of the enzyme from the NPs, the release profile was studied at the biologically relevant pHs of 7.4 (representing blood circulation) and 4.5 (lysosome).

**Figure 5:**
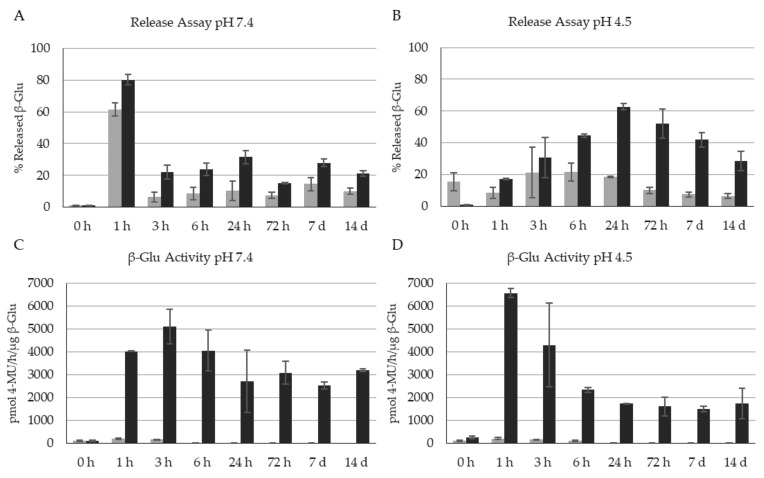
Quantification of the release and enzyme activity of β-glucosidase from the nanoparticles. Grey: control Solution 0 NPs (no Tween^®^ 20). Black: Solution 4 NPs (with Tween^®^ 20). (**A**) %Release of β-Glu at pH 7.4, (**B**) %Release of β-Glu at pH 4.5, (**C**) Activity of released β-Glu at pH 7.4, (**D**) Activity of released β-Glu at pH 4.5. Analysis of N = 3 separate NP formulations.

Unfortunately, a detailed analysis of the release kinetics using the Peppas model was not feasible due to the equilibrium of the release of enzyme from the NPs and the subsequent loss of enzyme activity in the supernatant, making it unquantifiable by HPLC, over time; therefore, we looked at each timepoint individually to measure both intact and active released enzyme. As previously seen in the article looking at β-Glu stabilization with BSA [56], enzyme loaded NPs with no stabilizer not only showed maximum release within 3 h at both pHs with a release of ~61% at pH 7.4 and 21.3% at pH 4.5 of the total enzyme content, but showed very little enzyme activity peaking at 200 pmol 4-MU/h/µg β-Glu. The difference in total amounts of recovered enzyme could be due to the instability of the enzyme in solution leading to lower quantification yields (Figure 5, Grey bars). The NPs formulated with Solution 4 showed very different results. A maximum release (62.7% at pH 4.5 and 80% at pH 7.4) was still observed within 24 h; however, two interesting observations were made. First, the released enzyme remained stable and measurable by HPLC even out to between 7 and 14 days (Figure 5A,B, black bars). Secondly, and in accordance with the first observation, the more stabilized enzyme retained activity at much higher levels and for longer times than the enzyme encapsulated alone. This is interesting because the results indicating low\poor complexation between Tween^®^ 20 and enzyme still led to stabilization in solution after being released in some way. One possible hypothesis for this still relates back to the stabilization of the NPs during formulation. More homogenous and constant NP formation could protect the enzyme from stresses such as solvent contact and sonication. These stresses could negatively impact the 3-D structure of the enzyme, leading it to be more susceptible to physical changes that lead to a loss of activity once released in biologically relevant conditions. Another hypothesis could be the “sacrificial lamb” effect: this would explain both the protection of the enzyme in solution under stress during formulation, but also explain as to why when the enzyme is released (presumably together with the incorporated Tween^®^ 20), it is protected long term and retains its activity.

**Figure 6:**
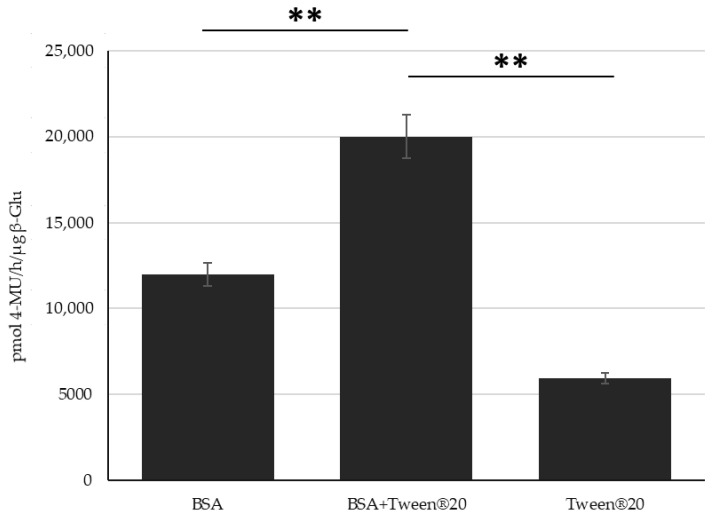
Combination Stabilization. Measurement of the β-Glu Activity in NPs formulated with: BSA:Enzyme, Tween^®^ 20:enzyme, or BSA:Tween^®^ 20:enzyme solution.. Statistical analysis was performed using the Student *t* test where, ** *p* < 0.01 and measured for N = 3 individual NP formulations of each type.

Due to the differences in interaction, loading efficiency and release profiles between Tween^®^ 20:enzyme solutions and BSA:enzyme complexes formulated into PLGA NPs, it was hypothesized that these two stabilizing molecules work by different (but still undefined) mechanisms. To this end it was tested to see if they could complement each other maintaining even higher levels of enzyme activity respective as each individually. Controls on stressed enzyme or stabilized enzyme under stress show this effect in solution (Figure S2) and therefore, studies during NP formulation were tested. NPs encapsulating BSA:β-Glu Complex (molar rapport 20:1), Tween^®^ 20:β-Glu Solution 4, and a BSA:Tween^®^ 20:β-Glu mixture solution (with the molar ratios of enzyme to BSA and Tween^®^ 20 equal to that of the individual formulations) were compared. NPs formulated with BSA:Enzyme, Tween^®^ 20:Enzyme, or BSA:Tween^®^ 20:Enzyme solutions were purified by centrifugation to remove any stabilizer or free enzyme in the supernatant. The intact NPs were then tested directly for the amount of β-Glu activity present. This was then normalized to the amount of enzyme encapsulated for each NP type based on an extraction and quantification of the amount of enzyme present. The combination of both stabilizers showed similar sizes (216 nm) and ζ-pot (−15 mV) as when formulated with only one of them. The %EE of BSA:Tween^®^:β-Glu was increased as previously seen with the BSA:β-Glu complex but not to the same extent (14 and 25%, respectively). Ultimately the combination of both stabilizers together in the formulation led to retention of a statistically higher enzyme activity in the NPs, an effect that appeared to be additive compared to NPs with the individual stabilizers (Figure 6).

## 4. Discussion

Hard to treat diseases are plaguing science at the moment with very few functional cures being approved to treat them. Nanomedicine has paved the way to creating new and improved treatments due to their ability to be targeted to certain organs, tissues, or cells, and to encapsulate and protect a wide variety of pharmaceutical molecules. Many diseases such as LSDs are caused by poor functioning or lack of enzymes which has led to a boom in research for ERT; however, enzyme administration has been a major bottleneck due to their delicate 3D structure required to maintain activity and lack of bioavailability when dosed in the blood [71,72,73]. Even with the improved prospects of encapsulating enzymes in NPs, the formulation process is known to deteriorate the enzyme, causing a loss of activity. For these reasons stabilizers are added to help maintain enzyme activity during encapsulation, but how they affect the enzyme and the formulations has not been described in detail in the literature. In fact, Tween^®^ is often used in enzyme particle formulations or during enzyme release assays without taking into consideration its potential stabilizing effect on the enzyme and its activity [74,75,76,77]. In this article, we expand on previous works looking at the complexation of BSA with β-Glu for improved activity by comparing the commercially available Tween^®^ series which has also been used in NP preparation.

Here polysorbates (more specifically Tween^®^ 20) demonstrated an efficient stabilizing effect of β-Glu in PLGA NPs. But it is very important to highlight the differences from the recently published work of the same type regarding BSA [56]. While BSA showed a direct interaction with the enzyme, Tween^®^ 20 did not. This highlights how the different stabilizers can affect an enzyme’s potential in different ways: BSA led to higher loading content and enzyme encapsulation (LC%–%EE) and the complexation also affected enzyme release, while Tween^®^ 20 did not show direct interaction with the enzyme, did not increase LC% or %EE to the same extent, and had no direct effect on the release kinetics. This strongly suggests a difference in mechanism where BSA directly stabilizes the enzyme during complexation prior formulation, while Tween^®^ has a much more global effect on the NP formation and stabilization throughout the formulative process. Characterizations of how stabilizers help maintaining high levels of enzyme activity also include studies on the oligomeric state of the enzyme. Even in recent years this topic is being published in the literature with high interest and not completely understood as results show differences in the oligomer state being dependent on numerous factors such as the stabilizer, inhibitor, and also origins of the β-Glu. Studies looking at β-Glu derived from *Aspergillus niger* demonstrate that surfactants can change the equilibrium versus the monomer increasing its activity by 60% [78]. On the other hand, β-Glu from *Spodoptera frugiperda* and almond demonstrate higher activity in the homodimer form of the enzyme [79,80]. Aim of this study was to analyze whether Tween^®^ had the same complexation rate of BSA, however advanced studies including X-ray crystallography, NMR, high-res electron microscopy or small angle neutron scattering are needed.

This difference between the two stabilizing mechanisms was further supported by the fact that these two effects could be complementary by using both stabilizers simultaneously to formulate enzyme loaded NPs with even more therapeutic potential. Having the knowledge and ability to combine stabilizers to increase enzyme loading into the NPs while maintaining its activity is key to the future of NP based ERT. While enzyme offer the advantage of higher long-term product turnover, PLGA NPs in the literature often demonstrate low encapsulation rates as seen in this work (0.4 mg/100 mg NPs), and the loss of activity during formulation is one of biggest inhibiting factors for their successful use. This 10-fold increase from 0.4 to 3.9 mg/100 mg using BSA as a stabilizer in enzyme NPs is a notable difference. This is similar to the 8 fold increase in activity of Tween^®^ 20:β-Glu NPs compared to non-stabilized NPs (approximately 6000 pmol 4-MU/h/µg β-Glu compared to 800 respectively) even without the increase in %LC. While combining the two stabilizers did not lead to the expected 20-fold increase, it did lead to a substantially improved result over each stabilizer by itself, which could break through the barrier of successful NP based ERT: literature precedence suggests that even small but highly active enzyme doses can lead to pathology corrections in vivo, paving the way for NP based therapies that ensure the delivery of active enzyme in a more bio-compatible and therapeutic manner to.

These results compared to the BSA stabilization results introduce a very interesting topic in the field of NP enzyme delivery: enzyme stabilization must be characterized more completely. This is because the appropriate selection or mixture of multiple stabilizers with different mechanisms significantly improve NP based ERT pharmaceutics in different ways, i.e., binding, stabilizing, increasing loading efficiency, or increasing both NP and enzyme stabilization. This seems to be an obvious statement but one that is drastically missing from the field in the literature. Too often, these types of stabilizers are taken for granted and used without quantification or optimization. This stems directly from the fact that most of these compounds are known to help stabilize the NP formulation or enzyme in some manner but are used as general constituents and not considered as direct players. This is highlighted by numerous citations where the presence of these stabilizers will go undiscussed, uncharacterized/unquantified, or without demonstrating the mode in which it affords increased enzyme activity potential for therapeutic delivery, or even more grave, NP systems will often be tested without the proper controls lacking the stabilizer to show its importance in the system [31,34,68,69,70,81].

This more in-depth characterization and quantification of stabilizers in NP systems is important from a characterization and understanding standpoint but becomes even more relevant when thinking about its possible biological effects. Tween^®^ as a surfactant is known to have potential cell membrane destabilizing effects and cell toxicity. Therefore, when unquantified and uncontrolled, this stabilizer could have devastating effects upon translation to clinical use [82,83,84,85,86]. In this study, we demonstrated that NPs formulated with various rapports of Tween^®^ 20:β-Glu and increasing amounts of Tween^®^ 20 had a constant incorporation of 9 mg Tween^®^/100 mg of NPs. This is critical information because it ensures that both in vitro, where our standard NP doses arrive at a maximum of 100 µg (9 µg Tween^®^) per well of cells, and for in vivo mice studies, in which we dose up to 20 mg/kg of NPs (45 µg Tween^®^), the amount of Tween^®^ administered within the NPs is lower than literature values for polysorbate toxicity; however, while this low amount is good for evading toxicity, it is yet to be tested in vivo if this amount is enough to maintain the enhanced BBB crossing potential of NPs with a polysorbate shell, as also described in the literature [87,88].

## 5. Conclusions

The results reported in this research article, in conjunction with previous published enzyme stabilization results, highlight the need to characterize the effects of different additives when discussing enzyme delivery. This is because while certain combinations of stabilizers could lead to additive effects further increasing therapeutic potential, it is not certain if two stabilizers that have the same mode of stabilization will be complementary. Therefore, interest in the mode of action of these stabilizers found abundantly in the literature should not be taken for granted as is, and further studies are needed to find potential combined systems. These new systems, that have already been demonstrated to be able to efficiently deliver enzymes in vitro and in vivo, have the potential to offer a much more effective therapy which a much higher acceptance rate than the current aggressive and invasive ERT approaches to treat enzyme-based diseases.

## Figures and Tables

**Table 1:** Tween^®^ 20:β-Glucosidase solutions.

Tween^®^ 20:β-Glu* Solution n.	% *v*/*v* (Tween^®^ 20/Water) **	Tween^®^ 20 (µmol)	Tween^®^ 20:β-Glu (mol:mol)
0	0	0	0
1	0.5	2.2	60:1
2	5.0	22.4	605:1
3	10.0	44.8	1209:1
4	20.0	89.6	2419:1

* β-Glu was held constant in each solution mixture (5 mg, 37 nmol); ** Total volume of the aqueous phase (MilliQ water and Tween^®^ 20) was held constant at 500 µL.

**Table 2:** Effect of Tween^®^ Type Comparison on Nanoparticle Characteristics.

Sample †	Z-Average nm ± S.D.	PDI ± S.D.	ζ-Pot mV ± S.D.	Yield% ± S.D.	LC% ± S.D.	EE% ± S.D.
Empty NPs	190 ± 15	0.06 ± 0.01	−20 ± 3	85.1 ± 3.1	-	-
NPs β-Glu	199 ± 25	0.09 ± 0.02	−22 ± 6	82.5 ± 6.8	0.4 ± 0.1	3.9 ± 1.5
NPs_Tween^®^ 20:β-Glu	198 ± 33	0.09 ± 0.06	−16 ± 7	75.9 ± 2.3	0.9 ± 0.1	7.8 ± 1.9 *
NPs_Tween^®^ 60:β-Glu	177 ± 44	0.05 ± 0.04	−23 ± 7	78.2 ± 1.8	0.8 ± 0.1	5.6 ± 2.6
NPs_Tween^®^ 80:β-Glu	208 ± 23	0.22 ± 0.03	−20 ± 4	72.7 ± 5.4	0.5 ± 0.1	5.4 ± 2.2

† the ratio of Tween^®^:β-Glu was held constant, mol:mol 2248:1; * statistically significant compared to NPs β-Glu, *p* = 0.049.

**Table 3:** Characteristics of Tween^®^ 20:β-Glucosidase Solutions.

Tween^®^ 20:β-Glu Solution n.	Z-Average nm ± S.D.	PDI ± S.D.	Peak 1 nm ± S.D. (% ± S.D.)	Peak 2 nm ± S.D. (% ± S.D.)	Peak 3 nm ± S.D. (% ± S.D.)	ζ-Pot mV ± S.D.
0	792 ± 27	0.65 ± 0.11	78 ± 12 (80 ± 4)	144 ± 45 (15 ± 5)	-	−2.21 ± 4
1	932 ± 225	0.79 ± 0.06	564 ± 200 (73 ± 11)	1185 ± 3120 (20 ± 3)	602 ± 915 (5 ± 3)	2.27 ± 3
2	636 ± 23	0.71 ± 0.09	521 ± 275 (60 ± 5)	36 ± 46 (38 ± 8)	3470 ± 2453 (2 ± 2)	2.12 ± 2
3	114 ± 60	0.35 ± 0.09	9 ± 1 (65 ± 7)	529 ± 11 (34 ± 7)	5125 ± 191 (8 ± 4)	2.13 ± 1
4	116 ± 47	0.49 ± 0.14	281 ± 470 (59 ± 8)	936 ± 935 (30 ± 9)	3218 ± 1697 (10 ± 8)	2.14 ± 1

**Table 4:** Physical characterization and loading content of Tween^®^ 20:β-Glucosidase solutions in PLGA NPs.

	Z-Average nm ± S.D.	PDI ± S.D.	AFM Diameter nm ± S.D.	ζ-Pot mV ± S.D.	Yield% ± S.D.	LC% ± S.D.	EE% ± S.D.
Empty NPs	190 ± 15	0.06 ± 0.01	320 ± 47	−20 ± 3	85.1 ± 3.1	-	-
NPs_Solution0	199 ± 25	0.09 ± 0.02	311 ± 69	−22 ± 6	82.5 ± 6.8	0.4 ± 0.1	3.9 ± 1.5
NPs_Solution1	185 ± 24	0.08 ± 0.03	173 ± 41	−16 ± 5	72.7 ± 5.5	1.1 ± 0.0	9.7 ± 1.2
NPs_Solution2	188 ± 13	0.08 ± 0.01	142 ± 39	−16 ± 10	73.9 ± 4.3	1.0 ± 0.1	11.1 ± 1.2
NPs_Solution3	191 ± 23	0.08 ± 0.02	150 ± 26	−16 ± 7	81.8 ± 3.6	0.8 ± 0.1	8.4 ± 4.0
NPs_Solution4	198 ± 33	0.09 ± 0.06	270 ± 31	−16 ± 7	75.9 ± 2.3	0.9 ± 0.1	7.8 ± 2.5

**Table 5:** Quantification of Tween^®^ 20 content in Nanoparticles formulated with solutions 1–4.

Samples	Tween^®^ 20 Content mg Tween^®^ 20/100 mg NPs	β-Glu Content mg β-Glu/100 mg NPs	Tween^®^ 20:β-Glu mol/mol
NPs_Solution1	9.4 ± 0.3	1.1 ± 0.0	844
NPs_Solution2	9.3 ± 0.2	1.0 ± 0.1	872
NPs_Solution3	9.3 ± 0.1	0.8 ± 0.1	1233
NPs_Solution4	9.7 ± 0.6	0.9 ± 0.1	1560

## Data Availability

The raw data are available upon request from the corresponding author.

## Supplementary Materials

The following are available online at https://www.mdpi.com/article/10.3390/nano11112946/s1, Figure S1: HPLC Quantification. Representative Chromatograms for the quantification of (**A**) β-Glucosidase and (**B**) Tween^®^ 20, Figure S2: β-Glucosidase Activity Controls. Control samples of the β-Glucosidase activity in solution or after a mock formulation process (including organic solvents, sonication, and centrifugation) with or without the presence of each stabilizer, Figure S3: AFM images of the different Nanoparticle preparations. (**A**) NPs PLGA:Solution0; (**B**) NPs PLGA:Solution1; (**C**) NPs PLGA:Solution2; (**D**) NPs PLGA:Solution3; (**E**) NPs PLGA:Solution4. Note: all formula-tions contain 5 mg β-Glu, Table S1: List of the abbreviations used in the main text.

## References

[1] Duskey J.T., Belletti D., Pederzoli F., Vandelli M.A., Forni F., Ruozi B., Tosi G. (2017). Current Strategies for the Delivery of Therapeutic Proteins and Enzymes to Treat Brain Disorders. Int. Rev. Neurobiol..

[2] Rigon L., Salvalaio M., Pederzoli F., Legnini E., Duskey J.T., D’Avanzo F., De Filippis C., Ruozi B., Marin O., Vandelli M.A. (2019). Targeting Brain Disease in MPSII: Preclinical Evaluation of IDS-Loaded PLGA Nanoparticles. Int. J. Mol. Sci..

[3] Mulvihill J.J., Cunnane E.M., Ross A.M., Duskey J.T., Tosi G., Grabrucker A.M. (2020). Drug Delivery across the Blood–Brain Barrier: Recent Advances in the Use of Nanocarriers. Nanomedicine.

[4] Tosi G., Duskey J.T., Kreuter J. (2020). Nanoparticles as Carriers for Drug Delivery of Macromolecules across the Blood-Brain Barrier. Expert Opin. Drug Deliv..

[5] Li M. (2018). Enzyme Replacement Therapy: A Review and Its Role in Treating Lysosomal Storage Diseases. Pediatric Ann..

[6] Eisengart J.B., Jarnes J., Ahmed A., Nestrasil I., Ziegler R., Delaney K., Shapiro E., Whitley C. (2017). Long-Term Cognitive and Somatic Outcomes of Enzyme Replacement Therapy in Untransplanted Hurler Syndrome. Mol. Genet. Metab. Rep..

[7] Gaffke L., Pierzynowska K., Piotrowska E., Węgrzyn G. (2018). How Close Are We to Therapies for Sanfilippo Disease?. Metab. Brain Dis..

[8] Wiseman J.A., Meng Y., Nemtsova Y., Matteson P.G., Millonig J.H., Moore D.F., Sleat D.E., Lobel P. (2017). Chronic Enzyme Replacement to the Brain of a Late Infantile Neuronal Ceroid Lipofuscinosis Mouse Has Differential Effects on Phenotypes of Disease. Mol. Ther.—Methods Clin. Dev..

[9] Solovyeva V.V., Shaimardanova A.A., Chulpanova D.S., Kitaeva K.V., Chakrabarti L., Rizvanov A.A. (2018). New Approaches to Tay-Sachs Disease Therapy. Front. Physiol.

[10] Kumari A., Yadav S.K., Yadav S.C. (2010). Biodegradable Polymeric Nanoparticles Based Drug Delivery Systems. Colloids Surf. B Biointerfaces.

[11] Wraith J.E. (2006). Limitations of Enzyme Replacement Therapy: Current and Future. J. Inherit. Metab. Dis..

[12] Concolino D., Deodato F., Parini R. (2018). Enzyme Replacement Therapy: Efficacy and Limitations. Ital. J. Pediatrics.

[13] Ries M. (2017). Enzyme Replacement Therapy and beyond—in Memoriam Roscoe O. Brady, M.D. (1923–2016). J. Inherit. Metab. Dis..

[14] Nelemans L.C., Gurevich L. (2020). Drug Delivery with Polymeric Nanocarriers—Cellular Uptake Mechanisms. Materials.

[15] Alven S., Aderibigbe B.A. (2020). Efficacy of Polymer-Based Nanocarriers for Co-Delivery of Curcumin and Selected Anticancer Drugs. Nanomaterials.

[16] Abasian P., Ghanavati S., Rahebi S., Khorasani S.N., Khalili S. (2020). Polymeric Nanocarriers in Targeted Drug Delivery Systems: A Review. Polym. Adv. Technol..

[17] Venditti I. (2019). Morphologies and Functionalities of Polymeric Nanocarriers as Chemical Tools for Drug Delivery: A Review. J. King Saud Univ.-Sci..

[18] Avramović N., Mandić B., Savić-Radojević A., Simić T. (2020). Polymeric Nanocarriers of Drug Delivery Systems in Cancer Therapy. Pharmaceutics.

[19] Duskey J.T., Baraldi C., Gamberini M.C., Ottonelli I., Da Ros F., Tosi G., Forni F., Vandelli M.A., Ruozi B. (2020). Investigating Novel Syntheses of a Series of Unique Hybrid PLGA-Chitosan Polymers for Potential Therapeutic Delivery Applications. Polymers.

[20] Oddone N., Boury F., Garcion E., Grabrucker A.M., Martinez M.C., Da Ros F., Janaszewska A., Forni F., Vandelli M.A., Tosi G. (2020). Synthesis, Characterization, and In Vitro Studies of an Reactive Oxygen Species (ROS)-Responsive Methoxy Polyethylene Glycol-Thioketal-Melphalan Prodrug for Glioblastoma Treatment. Front. Pharmacol..

[21] Belletti D., Riva G., Luppi M., Tosi G., Forni F., Vandelli M.A., Ruozi B., Pederzoli F. (2017). Anticancer Drug-Loaded Quantum Dots Engineered Polymeric Nanoparticles: Diagnosis/Therapy Combined Approach. Eur. J. Pharm. Sci..

[22] Dinu I.A., Duskey J.T., Car A., Palivan C.G., Meier W. (2016). Engineered Non-Toxic Cationic Nanocarriers with Photo-Triggered Slow-Release Properties. Polym. Chem..

[23] Birolini G., Valenza M., Ottonelli I., Passoni A., Favagrossa M., Duskey J.T., Bombaci M., Vandelli M.A., Colombo L., Bagnati R. (2021). Insights into Kinetics, Release, and Behavioral Effects of Brain-Targeted Hybrid Nanoparticles for Cholesterol Delivery in Huntington’s Disease. J. Control. Release.

[24] Musumeci T., Bonaccorso A., Carbone C., Impallomeni G., Ballistreri A., Duskey J.T., Puglisi G., Pignatello R. (2020). Development and Biocompatibility Assessments of Poly(3-Hydroxybutyrate-Co-ε-Caprolactone) Microparticles for Diclofenac Sodium Delivery. J. Drug Deliv. Sci. Technol..

[25] Pederzoli F., Ruozi B., Duskey J., Hagmeyer S., Sauer A.K., Grabrucker S., Coelho R., Oddone N., Ottonelli I., Daini E. (2019). Nanomedicine against Aβ Aggregation by β–Sheet Breaker Peptide Delivery: In Vitro Evidence. Pharmaceutics.

[26] Puiggalí-Jou A., del Valle L.J., Alemán C. (2020). Encapsulation and Storage of Therapeutic Fibrin-Homing Peptides Using Conducting Polymer Nanoparticles for Programmed Release by Electrical Stimulation. ACS Biomater. Sci. Eng..

[27] Du A.W., Stenzel M.H. (2014). Drug Carriers for the Delivery of Therapeutic Peptides. Biomacromolecules.

[28] Elsabahy M., Song Y., Eissa N.G., Khan S., Hamad M.A., Wooley K.L. (2021). Morphologic Design of Sugar-Based Polymer Nanoparticles for Delivery of Antidiabetic Peptides. J. Control. Release.

[29] Tosi G., Pederzoli F., Belletti D., Vandelli M.A., Forni F., Duskey J.T., Ruozi B. (2019). Nanomedicine in Alzheimer’s Disease: Amyloid Beta Targeting Strategy. Prog. Brain Res..

[30] Liu J., Postupalenko V., Duskey J.T., Palivan C.G., Meier W. (2015). PH-Triggered Reversible Multiple Protein–Polymer Conjugation Based on Molecular Recognition. J. Phys. Chem. B.

[31] Cózar-Bernal M.J., Holgado M.A., Arias J.L., Muñoz-Rubio I., Martín-Banderas L., Álvarez-Fuentes J., Fernández-Arévalo M. (2011). Insulin-Loaded PLGA Microparticles: Flow Focusing versus Double Emulsion/Solvent Evaporation. J. Microencapsul..

[32] Sigg S.J., Postupalenko V., Duskey J.T., Palivan C.G., Meier W. (2016). Stimuli-Responsive Codelivery of Oligonucleotides and Drugs by Self-Assembled Peptide Nanoparticles. Biomacromolecules.

[33] Nussbaumer M.G., Duskey J.T., Rother M., Renggli K., Chami M., Bruns N. (2016). Chaperonin–Dendrimer Conjugates for SiRNA Delivery. Adv. Sci..

[34] Zou W., Liu C., Chen Z., Zhang N. (2009). Preparation and Characterization of Cationic PLA-PEG Nanoparticles for Delivery of Plasmid DNA. Nanoscale Res. Lett..

[35] Muro S. (2010). New Biotechnological and Nanomedicine Strategies for Treatment of Lysosomal Storage Disorders. Wiley Interdiscip. Rev. Nanomed. Nanobiotechnol..

[36] Martinez N.Y., Andrade P.F., Durán N., Cavalitto S. (2017). Development of Double Emulsion Nanoparticles for the Encapsulation of Bovine Serum Albumin. Colloids Surf. B Biointerfaces.

[37] Jahangiri A., Barghi L. (2018). Polymeric Nanoparticles: Review of Synthesis Methods and Applications in Drug Delivery. J. Adv. Chem. Pharm. Mater. (JACPM).

[38] Mohanty S., Panda S., Purohit D., Si S.C. (2019). A Comprehensive Review on PLGA-Based Nanoparticles Used for Rheumatoid Arthritis. Rese. J. Pharm. Technol..

[39] Xu Y., Kim C.-S., Saylor D.M., Koo D. (2017). Polymer Degradation and Drug Delivery in PLGA-Based Drug–Polymer Applications: A Review of Experiments and Theories. J. Biomed. Mater. Res. Part B Appl. Biomater..

[40] Yaghoobi N., Faridi Majidi R., Faramarzi M.A., Baharifar H., Amani A. (2017). Preparation, Optimization and Activity Evaluation of PLGA/Streptokinase Nanoparticles Using Electrospray. Adv. Pharm. Bull..

[41] Hasanpour A., Esmaeili F., Hosseini H., Amani A. (2021). Use of MPEG-PLGA Nanoparticles to Improve Bioactivity and Hemocompatibility of Streptokinase: In-Vitro and in-Vivo Studies. Mater. Sci. Eng. C.

[42] Ding D., Zhu Q. (2018). Recent Advances of PLGA Micro/Nanoparticles for the Delivery of Biomacromolecular Therapeutics. Mater. Sci. Eng. C.

[43] Mohammadpour F., Hadizadeh F., Tafaghodi M., Sadri K., Mohammadpour A.H., Kalani M.R., Gholami L., Mahmoudi A., Chamani J. (2019). Preparation, in Vitro and in Vivo Evaluation of PLGA/Chitosan Based Nano-Complex as a Novel Insulin Delivery Formulation. Int. J. Pharm..

[44] Kaplan M.A., Sergienko K.V., Kolmakova A.A., Konushkin S.V., Baikin A.S., Kolmakov A.G., Sevostyanov M.A., Kulikov A.V., Ivanov V.E., Belosludtsev K.N. (2020). Development of a Biocompatible PLGA Polymers Capable to Release Thrombolytic Enzyme Prourokinase. J. Biomater. Sci. Polym. Ed..

[45] Salvalaio M., Rigon L., Belletti D., D’Avanzo F., Pederzoli F., Ruozi B., Marin O., Vandelli M.A., Forni F., Scarpa M. (2016). Targeted Polymeric Nanoparticles for Brain Delivery of High Molecular Weight Molecules in Lysosomal Storage Disorders. PLoS ONE.

[46] Yun X., Maximov V.D., Yu J., Zhu G., Vertegel A.A., Kindy M.S. (2013). Nanoparticles for Targeted Delivery of Antioxidant Enzymes to the Brain after Cerebral Ischemia and Reperfusion Injury. J. Cereb Blood Flow Metab..

[47] Schuster T., Mühlstein A., Yaghootfam C., Maksimenko O., Shipulo E., Gelperina S., Kreuter J., Gieselmann V., Matzner U. (2017). Potential of Surfactant-Coated Nanoparticles to Improve Brain Delivery of Arylsulfatase A. J. Control. Release.

[48] Pérez C., Castellanos I.J., Costantino H.R., Al-Azzam W., Griebenow K. (2002). Recent Trends in Stabilizing Protein Structure upon Encapsulation and Release from Bioerodible Polymers. J. Pharm. Pharmacol..

[49] Primavessy D., Günday Türeli N., Schneider M. (2017). Influence of Different Stabilizers on the Encapsulation of Desmopressin Acetate into PLGA Nanoparticles. Eur. J. Pharm. Biopharm..

[50] Van de Weert M., Hennink W.E., Jiskoot W. (2000). Protein Instability in Poly(Lactic-Co-Glycolic Acid) Microparticles. Pharm. Res..

[51] Fonte P., Soares S., Sousa F., Costa A., Seabra V., Reis S., Sarmento B. (2014). Stability Study Perspective of the Effect of Freeze-Drying Using Cryoprotectants on the Structure of Insulin Loaded into PLGA Nanoparticles. Biomacromolecules.

[52] Taghipour B., Yakhchali M., Haririan I., Tamaddon A.M., Samani S.M. (2014). The Effects of Technical and Compositional Variables on the Size and Release Profile of Bovine Serum Albumin from PLGA Based Particulate Systems. Res. Pharm. Sci..

[53] Imamura K., Murai K., Korehisa T., Shimizu N., Yamahira R., Matsuura T., Tada H., Imanaka H., Ishida N., Nakanishi K. (2014). Characteristics of Sugar Surfactants in Stabilizing Proteins During Freeze–Thawing and Freeze–Drying. J. Pharm. Sci..

[54] Paillard-Giteau A., Tran V.T., Thomas O., Garric X., Coudane J., Marchal S., Chourpa I., Benoît J.P., Montero-Menei C.N., Venier-Julienne M.C. (2010). Effect of Various Additives and Polymers on Lysozyme Release from PLGA Microspheres Prepared by an s/o/w Emulsion Technique. Eur. J. Pharm. Biopharm..

[55] Rosa G.D., Iommelli R., La Rotonda M.I., Miro A., Quaglia F. (2000). Influence of the Co-Encapsulation of Different Non-Ionic Surfactants on the Properties of PLGA Insulin-Loaded Microspheres. J. Control. Release.

[56] Duskey J.T., da Ros F., Ottonelli I., Zambelli B., Vandelli M.A., Tosi G., Ruozi B. (2020). Enzyme Stability in Nanoparticle Preparations Part 1: Bovine Serum Albumin Improves Enzyme Function. Molecules.

[57] Saraswat M., Reddy P.Y., Muthenna P., Reddy G.B. (2008). Prevention of Non-Enzymic Glycation of Proteins by Dietary Agents: Prospects for Alleviating Diabetic Complications. Br. J. Nutr..

[58] Pirooznia N., Hasannia S., Lotfi A.S., Ghanei M. (2012). Encapsulation of Alpha-1 Antitrypsin in PLGA Nanoparticles: In Vitro Characterization as an Effective Aerosol Formulation in Pulmonary Diseases. J. Nanobiotechnol..

[59] Iwai J., Ogawa N., Nagase H., Endo T., Loftsson T., Ueda H. (2007). Effects of Various Cyclodextrins on the Stability of Freeze-Dried Lactate Dehydrogenase. J. Pharm. Sci..

[60] Osman R., Kan P.L., Awad G., Mortada N., EL-Shamy A.-E., Alpar O. (2011). Enhanced Properties of Discrete Pulmonary Deoxyribonuclease I (DNaseI) Loaded PLGA Nanoparticles during Encapsulation and Activity Determination. Int. J. Pharm..

[61] Atkins D.L., Magana J.R., Sproncken C.C.M., van Hest J.C.M., Voets I.K. (2021). Single Enzyme Nanoparticles with Improved Biocatalytic Activity through Protein Entrapment in a Surfactant Shell. Biomacromolecules.

[62] Hans M.L., Lowman A.M. (2002). Biodegradable Nanoparticles for Drug Delivery and Targeting. Curr. Opin. Solid State Mater. Sci..

[63] Astete C.E., Sabliov C.M. (2006). Synthesis and Characterization of PLGA Nanoparticles. J. Biomater. Sci. Polym. Ed..

[64] Arsiccio A., McCarty J., Pisano R., Shea J.-E. (2018). Effect of Surfactants on Surface-Induced Denaturation of Proteins: Evidence of an Orientation-Dependent Mechanism. J. Phys. Chem. B.

[65] Michelin K., Wajner A., Goulart L.S., Fachel Â.A., Pereira M.L.S., de Mello A.S., Souza F.T.S., Pires R.F., Giugliani R., Coelho J.C. (2004). Biochemical Study on β-Glucosidase in Individuals with Gaucher’s Disease and Normal Subjects. Clin. Chim. Acta.

[66] Belletti D., Grabrucker A.M., Pederzoli F., Menrath I., Vandelli M.A., Tosi G., Duskey T.J., Forni F., Ruozi B. (2018). Hybrid Nanoparticles as a New Technological Approach to Enhance the Delivery of Cholesterol into the Brain. Int. J. Pharm..

[67] Nayak V.S., Tan Z., Ihnat P.M., Russell R.J., Grace M.J. (2012). Evaporative Light Scattering Detection Based HPLC Method for the Determination of Polysorbate 80 in Therapeutic Protein Formulations. J. Chromatogr. Sci..

[68] Yoshii H., Buche F., Takeuchi N., Terrol C., Ohgawara M., Furuta T. (2008). Effects of Protein on Retention of ADH Enzyme Activity Encapsulated in Trehalose Matrices by Spray Drying. J. Food Eng..

[69] Bhatt P.C., Verma A., Al-Abbasi F.A., Anwar F., Kumar V., Panda B.P. (2017). Development of Surface-Engineered PLGA Nanoparticulate-Delivery System of Tet1-Conjugated Nattokinase Enzyme for Inhibition of Aβ40 Plaques in Alzheimer’s Disease. Int. J. Nanomed..

[70] Reddy M.K., Wu L., Kou W., Ghorpade A., Labhasetwar V. (2008). Superoxide Dismutase-Loaded PLGA Nanoparticles Protect Cultured Human Neurons Under Oxidative Stress. Appl. Biochem. Biotechnol..

[71] Parenti G., Pignata C., Vajro P., Salerno M. (2013). New Strategies for the Treatment of Lysosomal Storage Diseases (Review). Int. J. Mol. Med..

[72] Ratko T.A., Marbella A., Godfrey S., Aronson N. (2013). Enzyme-Replacement Therapies for Lysosomal Storage Diseases.

[73] Ullman J.C., Arguello A., Getz J.A., Bhalla A., Mahon C.S., Wang J., Giese T., Bedard C., Kim D.J., Blumenfeld J.R. (2020). Brain Delivery and Activity of a Lysosomal Enzyme Using a Blood-Brain Barrier Transport Vehicle in Mice. Sci. Transl. Med..

[74] Tang R., Kim C.S., Solfiell D.J., Rana S., Mout R., Velázquez-Delgado E.M., Chompoosor A., Jeong Y., Yan B., Zhu Z.-J. (2013). Direct Delivery of Functional Proteins and Enzymes to the Cytosol Using Nanoparticle-Stabilized Nanocapsules. ACS Nano.

[75] Johnson A.K., Zawadzka A.M., Deobald L.A., Crawford R.L., Paszczynski A.J. (2008). Novel Method for Immobilization of Enzymes to Magnetic Nanoparticles. J. Nanopart. Res..

[76] Naeem M., Kim W., Cao J., Jung Y., Yoo J.-W. (2014). Enzyme/PH Dual Sensitive Polymeric Nanoparticles for Targeted Drug Delivery to the Inflamed Colon. Colloids Surf. B Biointerfaces.

[77] Han C., Goodwine J., Romero N., Steck K.S., Sauer K., Doiron A. (2019). Enzyme-Encapsulating Polymeric Nanoparticles: A Potential Adjunctive Therapy in Pseudomonas Aeruginosa Biofilm-Associated Infection Treatment. Colloids Surf. B Biointerfaces.

[78] Seidel Z.P., Lee C.T. (2020). Enhanced Activity of the Cellulase Enzyme β-Glucosidase upon Addition of an Azobenzene-Based Surfactant. ACS Sustain. Chem. Eng..

[79] Otsuka F.A.M., Chagas R.S., Almeida V.M., Marana S.R. (2020). Homodimerization of a Glycoside Hydrolase Family GH1 β-Glucosidase Suggests Distinct Activity of Enzyme Different States. Protein Sci..

[80] Caramia S., Gatius A.G.M., dal Piaz F., Gaja D., Hochkoeppler A. (2017). Dual Role of Imidazole as Activator/Inhibitor of Sweet Almond (Prunus Dulcis) β-Glucosidase. Biochem. Biophys. Rep..

[81] Yusuf M., Khan M., Alrobaian M.M., Alghamdi S.A., Warsi M.H., Sultana S., Khan R.A. (2021). Brain Targeted Polysorbate-80 Coated PLGA Thymoquinone Nanoparticles for the Treatment of Alzheimer’s Disease, with Biomechanistic Insights. J. Drug Deliv. Sci. Technol..

[82] Hodaei D., Baradaran B., Valizadeh H., Mohammadnejad L., Zakeri P. (2014). The Effect of Tween Excipients on Expression and Activity of P-Glycoprotein in Caco-2 Cells. Pharm. Ind..

[83] Yang S., Liu J., Chen Y., Jiang J. (2012). Reversal Effect of Tween-20 on Multidrug Resistance in Tumor Cells in Vitro. Biomed. Pharmacother..

[84] Dimitrijevic D., Shaw A.J., Florence A.T. (2000). Effects of Some Non-Ionic Surfactants on Transepithelial Permeability in Caco-2 Cells. J. Pharm. Pharmacol..

[85] Scherließ R. (2011). The MTT Assay as Tool to Evaluate and Compare Excipient Toxicity in Vitro on Respiratory Epithelial Cells. Int. J. Pharm..

[86] Farkas W.R., Lorch V., Conover W.R., Al-Ansari H.M.H., Abney L.K., Painter P.C., Reyniers J.P., Congdon C.C. (1991). Polysorbate Toxicity in Neonatal Rats and Mice. Pharmacol. Toxicol..

[87] Chaturvedi M., Molino Y., Sreedhar B., Khrestchatisky M., Kaczmarek L. (2014). Tissue Inhibitor of Matrix Metalloproteinases-1 Loaded Poly(Lactic-Co-Glycolic Acid) Nanoparticles for Delivery across the Blood–Brain Barrier. Int J. Nanomed..

[88] Gelperina S., Maksimenko O., Khalansky A., Vanchugova L., Shipulo E., Abbasova K., Berdiev R., Wohlfart S., Chepurnova N., Kreuter J. (2010). Drug Delivery to the Brain Using Surfactant-Coated Poly(Lactide-Co-Glycolide) Nanoparticles: Influence of the Formulation Parameters. Eur. J. Pharm. Biopharm..

