# Supplementary material for: Tween® Preserves Enzyme Activity and Stability in PLGA Nanoparticles
Source: Nanomaterials (Basel). 2021 Nov 3;11(11):2946. doi: 10.3390/nano11112946 (PMC8625811; doi:10.3390/nano11112946)
Supplement: Supplementary file 1 [file nanomaterials-11-02946-s001.zip › nanomaterials-1382363-supplementary.pdf]

nanomaterials

Supplementary Materials

Tween® Preserves Enzyme Activity and Stability in PLGA Nanoparticles

Jason Thomas Duskey [1], Ilaria Ottonelli [1,2], Arianna Rinaldi [1,2], Irene Parmeggiani [1], Barbara Zambelli [3], Leon Z. Wang [4], Robert K. Prud'homme [4], Maria Angela Vandelli [1], Giovanni Tosi [1] and Barbara Ruozi [1,*]

[1] Te.Far.T.I.-Nanotech Lab, Department of Life Sciences, University of Modena and Reggio Emilia, 41125 Modena, Italy; jasonthomas.duskey@unimore.it (J.T.D.); ilaria.ottonelli@unimore.it (I.O.); arianna.rinaldi@unimore.it (A.R.); irene.parmeggiani@unimore.it (I.P.); mariaangela.vandelli@unimore.it (M.A.V.); gtosi@unimore.it (G.T.)
[2] Clinical and Experimental Medicine PhD Program, Department of Biomedical, Metabolic and Neural Sciences, University of Modena and Reggio Emilia, 41125 Modena, Italy
[3] Laboratory of Bioinorganic Chemistry, Department of Pharmacy and Biotechnology, University of Bologna, 40127 Bologna, Italy; barbara.zambelli@unibo.it
[4] Department of Chemical and Biological Engineering, Princeton University, Princeton, NJ 08544, USA; lzwang@princeton.edu (L.Z.W.); Prudhomm@princeton.edu (R.K.P.)
* Correspondence: barbara.ruozi@unimore.it; Tel.: +39-059-205-8562

Table S1. List of the abbreviations used in the main text

- % = percentage
- °C = Degrees Celsius
- AFM = Atomic Force Microscopy
- B-Glu = β-Glucosidase
- BBB = Blood Brain Barrier
- BSA = Bovine Serum Albumin
- CNS = Central Nervous System
- DCM = dichloromethane
- EE = entrapment efficiency
- ELSD = Evaporative Light Scattering Detector
- ERT = Enzyme Replacement Therapy
- GoF = Goodness of Fit
- h = hours
- ITC = Isothermal titration calorimetry
- J = joules
- K = kelvin
- KA = binding constant
- kHz = kilohertz
- LC = loading capacity
- ln = logarithm
- LSDs = Lysosomal Storage Disorders
- mg = milligram
- mg/mL = milligram per milliliter
- min = minute
- mL/min = milliliter per minute
- mm/Hg = millimeters of mercury

- mM= millimolar
- mol = mole
- MU = 4-Methylumbelliferone
- nm = nanometer
- NP = Nanoparticle
- p = p-value
- PBS = Phosphate-buffered saline
- PCS = Photon Correlation Spectroscopy
- PDI= polydispersity index
- PLGA = Poly(D, L-lactide-co-glycolide)
- pmol = picomole
- PVA = poly(vinyl alcohol)
- R = gas constant (8.314 J $K^{-1}mol^{-1}$)
- RP-HPLC = Reversed-Phase High-Performance Liquid Chromatography
- rpm = revolutions per minute
- RT = room temperature
- s = second
- S.D. = standard deviation
- SEM-FEG = Scanning Electron Microscopy Field Emission Gun
- T = temperature in Kelvin
- VWD = Variable wavelength detector
- β-Glu = β-Glucosidase
- ΔG = Gibbs Free Energy
- ΔH = enthalpy change
- ζ-pot = zeta potential
- μg = microgram
- μg/mL = microgram per milliliter
- μL = microliter
- μM = micromolar

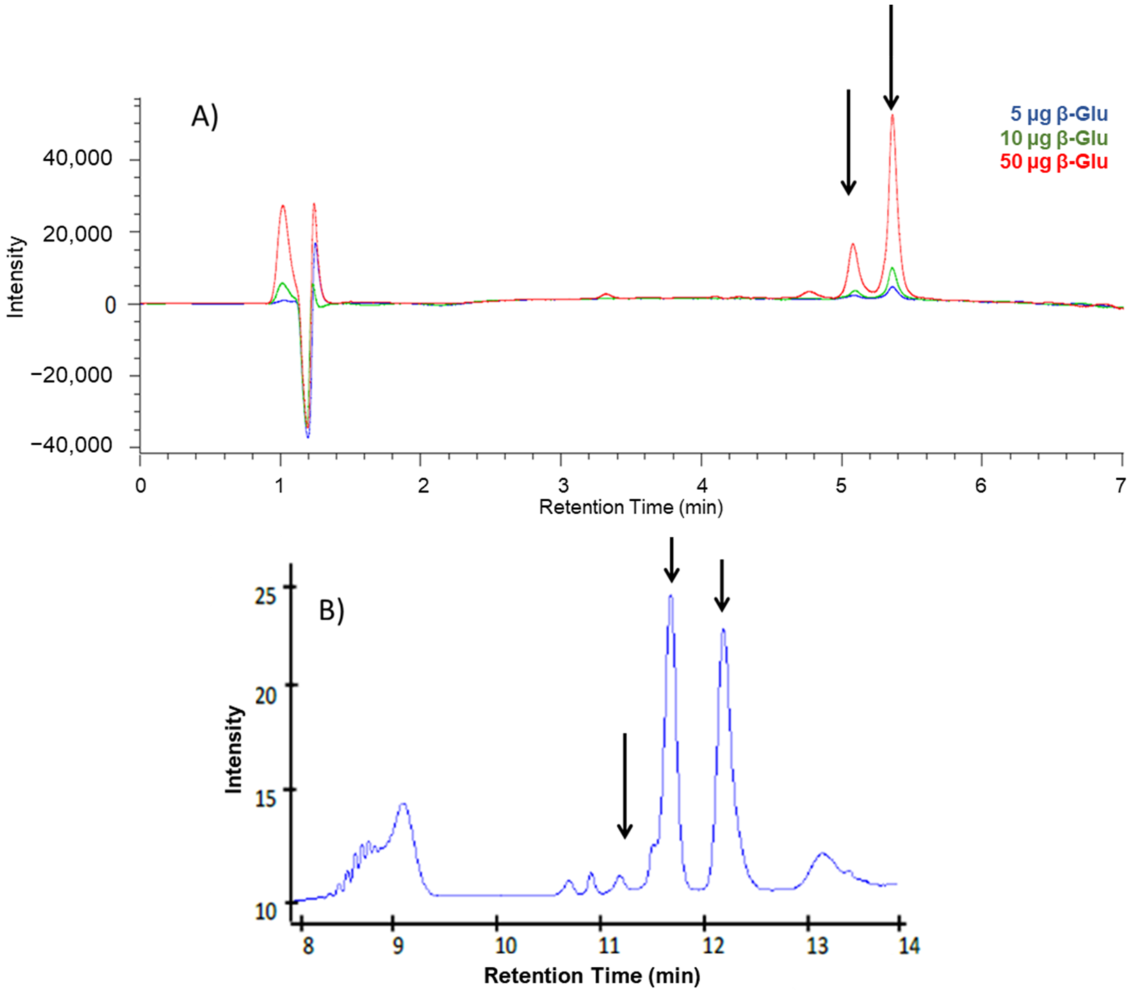

Figure S1. HPLC Quantification. Representative Chromatograms for the quantification of (**A**) β-Glucosidase and (**B**) Tween® 20.

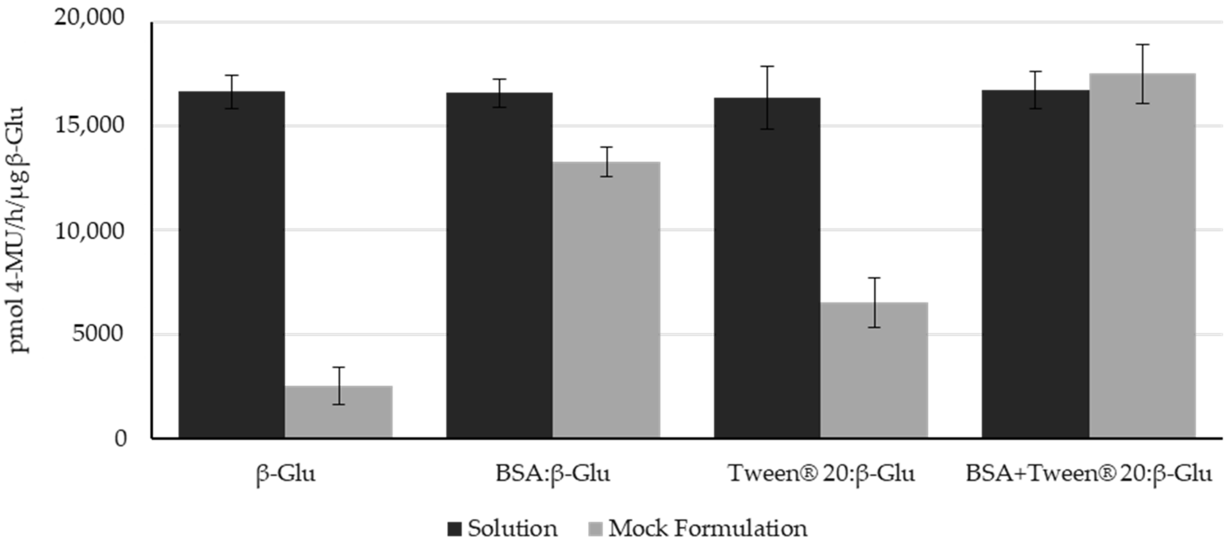

Figure S2. β-Glucosidase Activity Controls. Control samples of the β-Glucosidase activity in solution or after a mock formulation process (including organic solvents, sonication, and centrifugation) with or without the presence of each stabilizer.

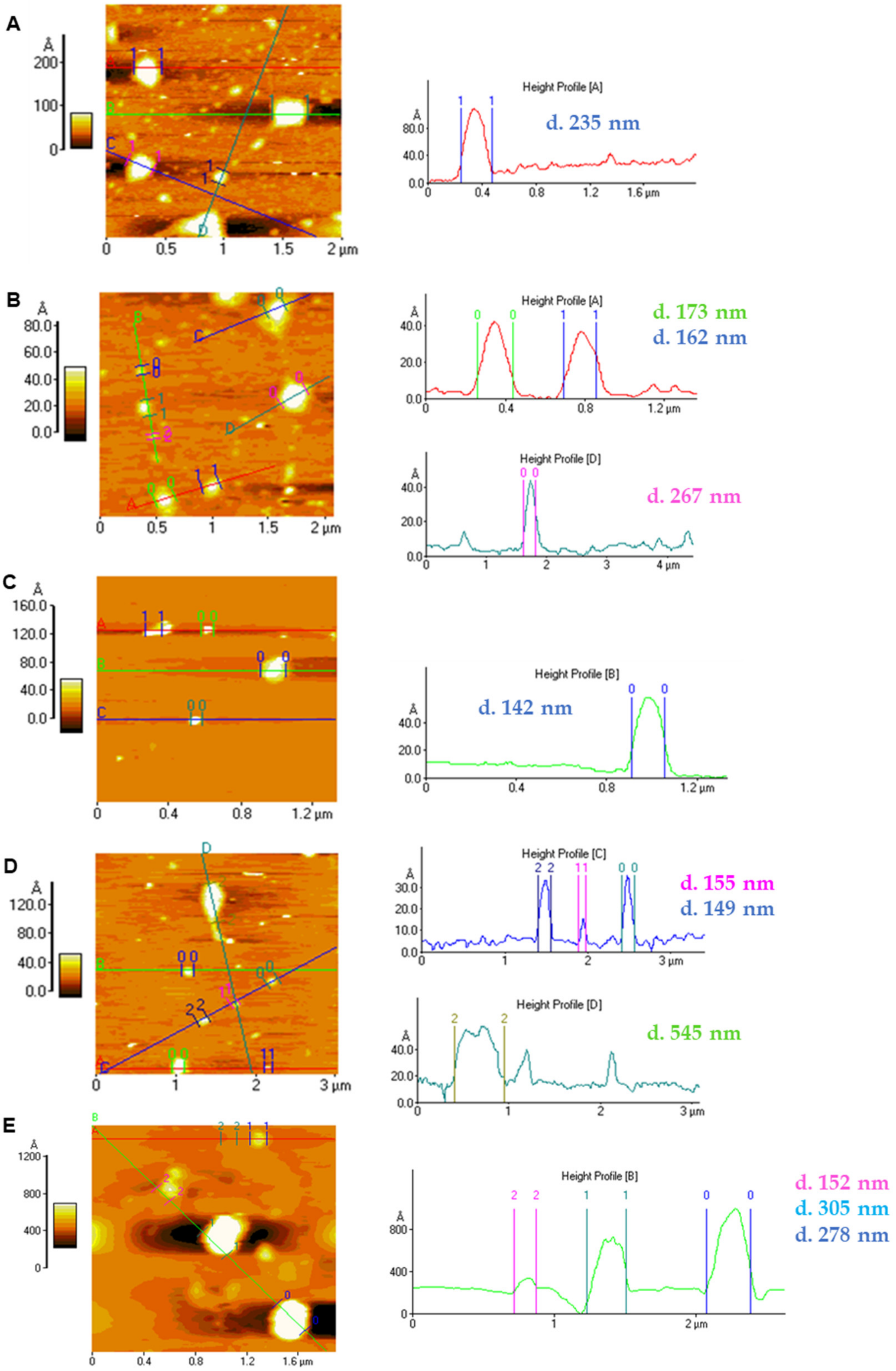

Figure S3. **AFM images of the different Nanoparticle preparations**. (**A**) NPs PLGA:Solution0; (**B**) NPs PLGA:Solution1; (**C**) NPs PLGA:Solution2; (**D**) NPs PLGA:Solution3; (**E**) NPs PLGA:Solution4. Note: all formulations contain 5 mg β-Glu.
